# Downregulation of DHRS9 expression in colorectal cancer tissues and its prognostic significance

**DOI:** 10.1007/s13277-015-3880-6

**Published:** 2015-08-08

**Authors:** Liang Hu, Hai-Yang Chen, Tao Han, Guang-Zhen Yang, Dan Feng, Chen-Ye Qi, Hui Gong, Yan-Xia Zhai, Qing-Ping Cai, Chun-Fang Gao

**Affiliations:** 1 Anal-Colorectal Surgery Institute, 150th Hospital of PLA, Luoyang, China; 2 Department of Oncology, 150th Hospital of PLA, Luoyang, China; 3 Department of Oncology, Cancer Center of People’s Liberation Army, General Hospital of Shenyang Military Region, Shenyang, China; 4 Department of Clinical Laboratory, 150th Hospital of PLA, Luoyang, China; 5 Department of Oncology, Changhai Hospital, Second Military Medical University, Shanghai, China; 6 Department of Gastrointestine Surgery, Changzheng Hospital, Second Military Medical University, Shanghai, China

**Keywords:** DHRS9, Colorectal cancer, Survival, Prognosis, Biomarker

## Abstract

Dehydrogenase/reductase (SDR family) member 9 (DHRS9) is aberrantly expressed in colorectal cancer (CRC), but its prognostic value is unknown. The aim of the work was to investigate the prognostic significance of DHRS9 expression in CRC. We found that DHRS9 was frequently downregulated in CRC clinical samples at both the messenger RNA (mRNA) and protein levels. Decreased expression of DHRS9 was significantly correlated with increased lymph node metastasis (*p* = 0.032), advanced tumor–node–metastasis (TNM) stage (*p* = 0.021), increased disease recurrence (*p* = 0.001), and death (*p* = 0.014). Kaplan–Meier analysis indicated that low DHRS9 expression predicted poor disease-free survival (*p* = 0.003) and disease-specific survival (*p* = 0.021). Cox multivariate analysis revealed that reduced expression of DHRS9 was an independent unfavorable prognostic indicator for CRC. Furthermore, combination of DHRS9 with TNM stage was a more powerful predictor of poor prognosis than either of the two parameters alone. Our results suggest that decreased expression of DHRS9 correlates with tumor progression and may serve as a potential prognostic biomarker in CRC.

## Introduction

Colorectal cancer (CRC) is one of the most commonly diagnosed cancers worldwide, with an estimated 1.4 million cases and 693,900 deaths occurring in 2012 [1]. Although the incidence of CRC in high-risk/high-income countries has varied over the past 20 years, it is increasing in certain historically low-risk countries, such as Spain and several countries in Eastern Europe and East Asia, which have been ascribed to changes in dietary patterns and risk factors toward a so-called Western lifestyle including unhealthy diet, obesity, and smoking [2–5]. In addition, increases in CRC mortality rates have been observed in countries that have more limited resources and increasing incidence [6–8]. At present, classification according to tumor–node–metastasis (TNM) stage provides valuable prognostic information and guides therapy decisions for CRC patients; nevertheless, clinical outcome differs greatly even among patients of the same TNM category [2]. Thus, there is an urgent need to search for valuable biomarkers to improve prognosis prediction and clinical outcome of patients with CRC.

Dehydrogenase/reductase (SDR family) member 9 (DHRS9), also known as retinol dehydrogenase L (RDHL), has been identified as a member of the short-chain dehydrogenases/reductase (SDR) family that converts retinol to retinal. Previous studies demonstrated that DHRS9 is involved in the biosynthesis of all-trans-retinoic acid (atRA), which displays important anti-tumor activity through inhibition of cell proliferation, induction of cell differentiation, and apoptosis and has been used in several cancer therapies including acute promyelocytic leukemia, squamous cell carcinoma, neuroblastoma, and hepatocellular carcinoma [9–13]. The enzymatic activity of DHRS9 was firstly characterized by Soref et al. in airway epitherial cells (referred to as hRDH-TBE in their publication) [14]. Later, Jette et al. reported that DHRS9 messenger RNA (mRNA) was primarily expressed in the colon, and its expression was low but detectable in the heart, spleen, placenta, and lung [15]. Using microarray and reverse transcriptase-PCR analysis, they demonstrated that DHRS9 was frequently and significantly downregulated in colon adenomas and carcinomas as compared with normal colon tissues. Consistent with the data from the adenoma and carcinoma samples, they found that colon-cancer-derived cell lines expressed low or undetectable levels of DHRS9 and displayed poor conversion of retinol to retinoic acid when compared to normal epithelial cells. Recently, a significant decreased expression of DHRS9 mRNA in CRC has also been confirmed by Kropotova et al. [10]. Mechanistic investigations revealed that reintroduction of the tumor suppressor adenomatous polyposis coli (APC) into the APC-deficient colon carcinoma cell line HT29 resulted in increased mRNA expression of DHRS9 via the transcription factor CDX2 [15]. In addition, DHRS9 could be induced by the lytic Epstein–Barr virus (EBV) immediate–early protein, BZLF1, in AGS gastric carcinoma cells containing the lytic form of EBV infection and EBV-positive Burkitt lymphoma cells [9]. Since lack of retinoic acid biosynthesis has been proposed as a mechanism contributing to the development of colon adenomas and carcinomas, we hypothesized that dysregulation of DHRS9 expression may be associated with aggressive clinical behavior of CRC. However, the clinical relevance of DHRS9 expression in CRC has not been investigated.

In the present study, we detected both the mRNA and the protein expression levels of DHRS9 in CRC clinical samples and further analyzed the correlation of DHRS9 expression with clinical features and with patient survival. Our results demonstrated that decreased expression of DHRS9 correlates with tumor progression and might serve as an independent unfavorable prognostic indicator for patients with CRC.

## Materials and methods

### Patients and follow-up

Formalin-fixed paraffin-embedded tissue specimens from 163 stages I–III CRC patients who received curative surgery in 150th Hospital of PLA (Luoyang, China) from April 2007 to September 2008 were retrieved for immunohistochemistry. The study cohort consisted of patients with CRC as confirmed by pathological analysis. Distribution of the continuous variables of the study cohort is listed in Table 1. Detailed clinicopathologic characteristics of the patients are listed in Table 2. The follow-up period was defined as the interval from the date of surgery to the date of death or last follow-up. The latest follow-up was updated in September 2014. Disease-specific survival (DSS) was defined as the interval from the date of surgery to the date that the patient died of CRC. Patients alive at the end of follow-up were censored. Disease-free survival (DFS) was defined as the interval from the date of surgery to the date of disease recurrence; if recurrence was not diagnosed, patients were censored on the date of death or last follow-up. Patients were excluded from the study cohorts with the following exclusion criteria: previously received any anti-cancer therapy; impaired heart, lung, liver, or kidney function; and previous malignant disease. TNM staging was classified according to the American Joint Committee on Cancer staging manual (seventh edition).

**Table 1:** Distribution of continuous variables of the study cohort (*n* = 163)

Variable	Median	Mean ± SEM	Range	Percentile	
				25th	75th
Age (years)	66.0	65.5 ± 0.9	31.0–91.0	58.0	74.0
Tumor size (cm)	5.0	5.1 ± 0.2	1.1–15.0	4.0	6.5
DSS (months)	75.0	57.9 ± 2.3	1.0–89.0	34.0	80.0
DFS (months)	62.0	50.7 ± 2.4	1.0–89.0	19.0	78.0

*SEM* standard error of the mean, *DSS* disease-specific survival, *DFS* disease-free survival

**Table 2:** Association between DHRS9 expression and clinicopathologic characteristics of CRC patients in the study cohort

Characteristics	No. of patients (%)	DHRS9 expression		
		Low (%)	High (%)	*p* value^a^
	(*n* = 163)	(*n* = 83)	(*n* = 80)	
Age (years)				0.573
<60	44 (27.0)	24 (28.9)	20 (25.0)	
≥60	119 (73.0)	59 (71.1)	60 (75.0)	
Sex				0.716
Female	71 (43.6)	35 (42.2)	36 (45.0)	
Male	92 (56.4)	48 (57.8)	44 (55.0)	
Tumor location				0.605
Rectum	68 (41.7)	33 (39.8)	35 (43.8)	
Colon	95 (58.3)	50 (60.2)	45 (56.2)	
Differentiation grade				0.780
Well	12 (7.3)	5 (6.0)	7 (8.8)	
Moderate	115 (70.6)	60 (72.3)	55 (68.8)	
Poor	36 (22.1)	18 (21.7)	18 (22.4)	
Tumor size (cm)				0.103
<5	67 (41.1)	29 (34.9)	38 (47.5)	
≥5	96 (58.9)	54 (65.1)	42 (52.5)	
Local invasion				0.860
T_1_–T_2_	17 (10.4)	9 (10.8)	8 (10.0)	
T_3_–T_4_	146 (89.6)	74 (89.2)	72 (90.0)	
Lymph node metastasis				**0.032**
N_0_	89 (54.6)	38 (45.8)	51 (63.8)	
N_1_	47 (28.8)	26 (31.3)	21 (26.2)	
N_2_	27 (16.6)	19 (22.9)	8 (10.0)	
TNM stage				**0.021**
I + II	89 (54.6)	38 (45.8)	51 (63.8)	
III	74 (45.4)	45 (54.2)	29 (36.2)	
Recurrence				**0.001**
No	76 (46.6)	28 (33.7)	48 (60.0)	
Yes	87 (53.4)	55 (66.3)	32 (40.0)	
Death				**0.014**
No	90 (55.2)	38 (45.8)	52 (65.0)	
Yes	73 (44.8)	45 (54.2)	28 (35.0)	

^a^Pearson chi-square test or Fisher exact test was used for comparison between subgroups. *Bold type* indicates statistical significance

Fresh-frozen CRC samples obtained from 58 stages I–III primary CRC patients who received curative surgery in 150th Hospital of PLA from April 2013 to September 2013 were used for quantitative polymerase chain reaction (qPCR) and Western blot analysis. Written informed consent was obtained from each patient, and this study was approved by the Ethical Committee of 150th Hospital of PLA.

### Real-time qPCR analysis

Real-time qPCR was performed as described previously [16]. Briefly, total RNAs were isolated from frozen specimens using TRIzol Reagent (Invitrogen). Reverse transcription (RT) was performed using RevertAidTM First Strand cDNA Synthesis Kit (Fermentas) according to the manufacturer’s instructions. After the RT reaction, the cDNA template was quantitated using real-time PCR technology (qPCR). qPCR was performed on ABI Prism 7500 Sequence Detection System with SYBR Premix Ex Taq^TM^ II (Takara) using the 2^−ΔΔCT^ method. Gene expression results were normalized by internal control β-actin. The primers used in this study are as follows: DHRS9 (NM_001142270.1) forward 5′-TTCCTTTGGCTGCTGACAGG-3′ and reverse 5′-ATTAGGAGGCCTAGCACCCA-3′, and β-actin forward 5′-AATCGTGCGTGACATTAAGGAG-3′ and reverse 5′-ACTGTGTTGG CGTACAGGTCTT-3′. Each sample was tested in duplicate.

### Western blot analysis

Western blotting was performed as described previously [17]. Briefly, tumor specimens were prepared in lysis buffer [Tris–HCl (20 mM), pH 7.4, NaCl (150 mM), glycerol (10 %), Nonidet P-40 (0.2 %), EDTA (1 mM), EGTA (1 mM), PMSF (1 mM), NaF (10 mM), aprotinin (5 mg/ml), leupeptin (20 mM), and sodium orthovanadate (1 mM)] and centrifuged at 12,000*g* for 30 min. Protein concentrations were measured using the BCA assay. Immunoblotting was performed using a primary antibody specific for DHRS9 (Abnova, H00010170-B01P), and immunocomplexes were incubated with goat anti-mouse fluorescein-conjugated secondary antibodies and then detected using an Odyssey fluorescence scanner (Li-Cor, Gene Company). β-actin was used as a loading control (Santa Cruz Biotechnology, sc-47778).

### Immunohistochemistry

Immunohistochemistry of paraffin-embedded tissue sections was performed as described previously [18]. Briefly, sections were deparaffinized and rehydrated. The endogenous peroxidase activity was blocked with 3 % H_2_O_2_ for 10 min. Antigens were retrieved with citrate buffer (10 mM, pH 6.0) for 15 min at 100 °C in a microwave oven. After blocking, the sections were incubated with a primary anti-DHRS9 Antibody (LifeSpan Biosciences, LS-C145077) with 1:200 dilution at 4 °C overnight in a moist chamber followed by incubation with an anti-rabbit peroxidase-conjugated secondary antibody (Santa Cruz) at room temperature for 30 min. Finally, the visualization signal was developed with diaminobenzidine (Dako), and the slides were counterstained with hematoxylin.

Stained sections were evaluated in a blinded manner without prior knowledge of the clinical data using the German immunoreactive score (IRS) as described previously [16, 19]. Briefly, staining intensity was graded as “0” (negative), “1” (weak), “2” (moderate), and “3” (strong); staining extent was graded as “0” (<5 %), “1” (5–25 %), “2” (25–50 %), “3” (50–75 %), or “4” (>75 %). Values of the staining intensity and the staining extent were multiplied as a final IRS of DHRS9 expression. The median IRS value of intratumoral DHRS9 expression was chosen as the cutoff for high and low DHRS9 expression levels based on a measure of heterogeneity according to the log-rank test with respect to DSS, as described previously [20, 21]. An IRS of ≥3 was used to define tumors with high DHRS9 expression, and an IRS of <3 was used to indicate tumors with low DHRS9 expression. Discrepancies in the IRS were resolved by discussing together with other pathologists to reach a consensus.

### Statistical analysis

Mann–Whitney *U* test was used to compare DHRS9 levels between groups. Pearson chi-square test or Fisher exact test was used to analyze the relationship between DHRS9 expression and clinical features. Kaplan–Meier analysis with log-rank test was used to compare patients’ survival between subgroups. The effect of each variable on survival was determined by the Cox multivariate regression analysis. All statistical analyses were carried out using SPSS PASW Statistics 18.0 software (SPSS, Inc., Chicago, IL), and *p* values <0.05 were considered to be statistically significant.

## Results

### Expression of DHRS9 in primary CRC tissues

**Fig. 1:**
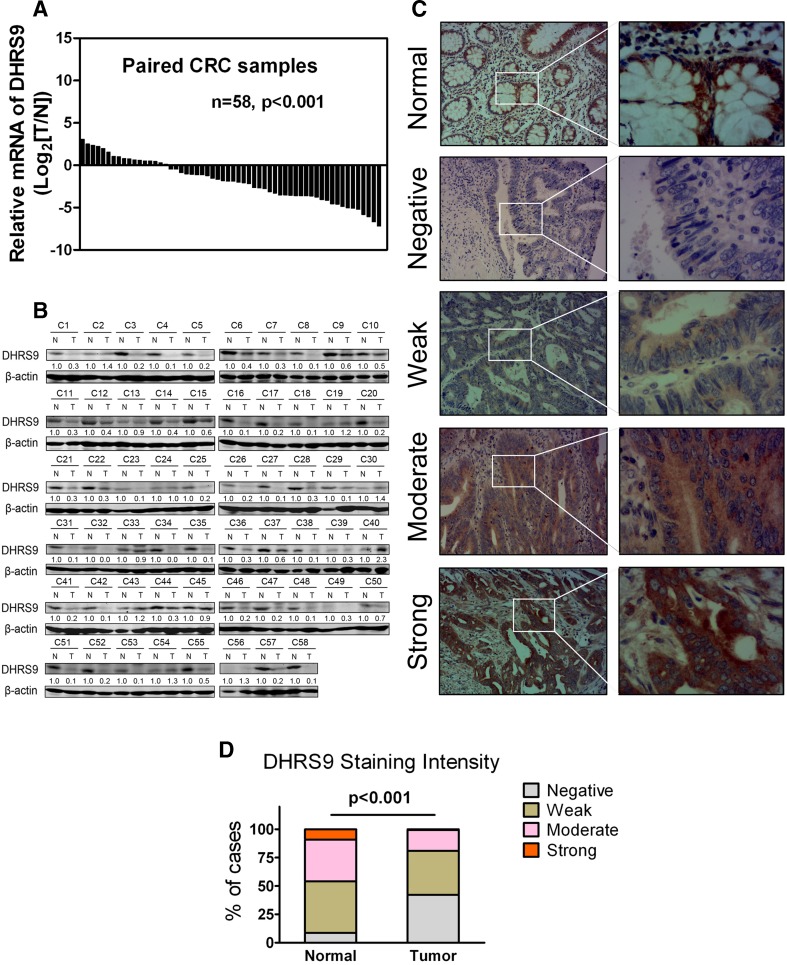
DHRS9 is frequently downregulated in CRC. **a** The expression levels of DHRS9 mRNA in 58 paired human primary CRC and corresponding adjacent normal mucosa specimens were determined by real-time qPCR methods. Gene expression results were normalized by internal control β-actin (*T*, tumor tissues; *N*, adjacent normal tissues). **b** Protein levels of DHRS9 in the same 58 paired CRC and corresponding adjacent normal specimens were determined by Western blot assay. β-actin was used as a loading control. The relative protein expression of DHRS9 was quantified and normalized to β-actin. Each N was arbitrarily designated 1.0. (*T*, tumor; *N*, adjacent normal tissues) **c** Representative immunohistochemical expression patterns of DHRS9 in cancerous and adjacent normal mucosa specimens are shown (magnification: *left panel*, ×100; *right panel*, ×400). **d** Percentage of cases with different staining intensity of DHRS9 in the tumor or adjacent normal tissues in the study cohort (*p* < 0.001)

The expression levels of DHRS9 mRNA in 58 paired human CRC tissues and corresponding adjacent normal mucosa tissues were quantified by real-time qPCR method. As shown in Fig. 1a, DHRS9 transcripts were significantly decreased in the cancerous tissues relative to the matched normal mucosa tissues (*p* < 0.001). In addition, Western blot assay was performed to determine the protein expression levels of DHRS9 on the same corresponding samples. Consistently, DHRS9 protein expression was also significantly lower in cancerous tissues than in adjacent normal counterparts (Fig. 1b).

We next detected the phenotypic expression patterns of DHRS9 protein in 163 paired paraffin-embedded CRC and corresponding adjacent normal mucosa specimens using immunohistochemistry. Representative staining of DHRS9 in CRC tissues is shown in Fig. 1c, and positive expression of DHRS9 was observed in the cytoplasm. DHRS9 protein expression was detected in 57.7 % (94/163) of the cancerous samples. Among them, 38.7 % (63/163), 18.4 % (30/163), and 0.6 % (1/163) of the cases show weak, moderate, and strong staining of DHRS9 protein, respectively. In contrast, 45.4 % (74/163), 36.8 % (60/163), and 9.2 % (15/163) of the adjacent normal mucosa specimens show weak, moderate, and strong staining of DHRS9, respectively (Fig. 1d). Thus, DHRS9 was frequently downregulated in CRC tissues at both the mRNA and protein levels.

### Correlation of DHRS9 expression with clinicopathologic features

The association between DHRS9 expression levels and clinicopathologic characteristics of CRC patients is summarized in Table 2. Reduced expression of DHRS9 was significantly correlated with increased lymph node metastasis (*p* = 0.032), advanced TNM stage (*p* = 0.021), increased disease recurrence (*p* = 0.001), and death (*p* = 0.014). However, there were no significant associations between DHRS9 expression and patient age (*p* = 0.573), sex (*p* = 0.716), tumor location (*p* = 0.605), tumor differentiation grade (*p* = 0.780), tumor size (*p* = 0.103), or local invasion (*p* = 0.860).

### Association of DHRS9 expression with prognosis of CRC patients

**Fig. 2:**
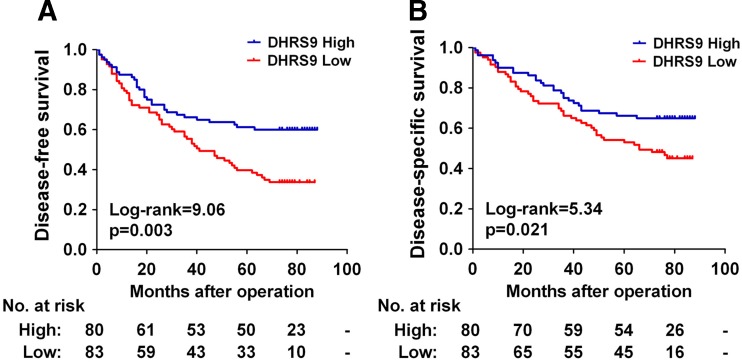
Kaplan–Meier survival analysis for CRC patients according to DHRS9 expression status. Kaplan–Meier curves for disease-free survival (**a**) or disease-specific survival (**b)** of the 163 CRC patients according to DHRS9 expression status (high or low expression). The *p* value was determined using the log-rank test. The absolute number of patients at risk in each subgroup is listed below

The 163 CRC patients were classified into high and low DHRS9 expression subgroups using its median IRS value as the cutoff point. The Kaplan–Meier analysis showed that patients in the low DHRS9 group had a significantly shorter disease-free survival (DFS) and worse disease-specific survival (DSS) than those in the high DHRS9 group (*p* = 0.003 and *p* = 0.021, respectively; Fig. 2a, b). The cumulative 5-year DFS and DSS rate was 61.3 and 66.3 % in patients with high-DHRS9 tumors, whereas it was only 39.8 and 53.0 % in those with low DHRS9 tumors, respectively.

To assess whether DHRS9 expression represents an independent prognostic indicator in CRC, the effect of each variable on survival was determined by the Cox regression analysis. Univariate analyses revealed that DHRS9 expression, TNM stage, and patient age were significantly associated with DSS, while DHRS9 expression and TNM stage were significantly associated with DFS (Table 3). The variables that significantly correlated with survival in the univariate analysis were further assessed by multivariate analysis. The results of the multivariate analysis confirmed that DHRS9 expression (hazard ratio (HR) = 1.715, 95 % confidence interval (95%CI) = 1.058–2.778, *p* = 0.029), TNM stage (HR = 3.047, 95%CI = 1.867–4.974, *p* < 0.001), and patient age (HR = 2.953, 95%CI = 1.592–5.477, *p* = 0.001) were independent prognostic factors for DSS. In addition, DHRS9 expression (HR = 1.767, 95%CI = 1.136–2.755, *p* = 0.012) and TNM stage (HR = 4.528, 95%CI = 2.830–7.244, *p* < 0.001) were independent prognostic factors for DFS (Table 3).

**Table 3:** Univariate and multivariate analyses of DHRS9 expression and patients’ survival

Variables	Categories	Univariate analysis			Multivariate analysis^a^		
		HR	95%CI	*p* value^b^	HR	95%CI	*p* value^b^
Disease-specific survival							
Age (years)	≥60/<60	1.996	1.095–3.636	**0.024**	2.953	1.592–5.477	**0.001**
Sex	Male/female	0.695	0.439–1.100	0.120			
Tumor location	Colon/rectum	0.947	0.595–1.506	0.818			
Tumor size (cm)	≥5/<5	1.037	0.649–1.657	0.880			
Differentiation grade	Poor/well + moderate	1.616	0.966–2.705	0.068			
TNM stage	III/I+ II	2.841	1.763–4.577	**<0.001**	3.047	1.867–4.974	**<0.001**
DHRS9 expression	Low/high	1.730	1.079–2.770	**0.023**	1.715	1.058–2.778	**0.029**
Disease-free survival							
Age (years)	≥60/<60	1.568	0.943–2.610	0.083			
Sex	Male/female	0.748	0.491–1.140	0.177			
Tumor location	Colon/rectum	0.935	0.611–1.430	0.756			
Tumor size (cm)	≥5/<5	1.017	0.662–1.561	0.939			
Differentiation grade	Poor/well + moderate	1.432	0.882–2.323	0.146			
TNM stage	III/I + II	4.093	2.587–6.475	**<0.001**	4.528	2.830–7.244	**<0.001**
DHRS9 expression	Low/high	1.927	1.245–2.985	**0.003**	1.767	1.136–2.755	**0.012**

*HR* hazard ratio, *95%CI* 95 % confidence interval, *TNM* tumor–node–metastasis

^a^Multivariate models were adjusted for age, sex, tumor location, tumor size, differentiation grade, and TNM stage

^b^Bold type indicates statistical significance

**Fig. 3:**
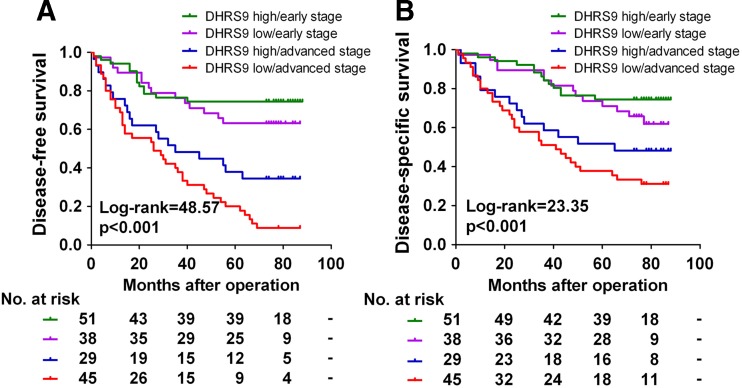
Kaplan–Meier survival analysis for CRC patients according to DHRS9 expression combined with TNM stage status. Kaplan–Meier curves for disease-free survival (**a**) or disease-specific survival (**b**) of the 163 CRC patients according to DHRS9 expression status (high or low expression) combined with TNM stage status (early or advanced stage). The *p* value was determined using the log-rank test. The absolute number of patients at risk in each subgroup is listed below

Since either decreased DHRS9 expression or advanced TNM stage predicts a poor prognosis of patients with CRC, we then observed the combination of DHRS9 expression and TNM stage as a predictor of clinical outcome. Importantly, the combination of these two parameters provided an improved prognostic value in comparison with the evaluation of either the TNM stage or DHRS9 expression alone (Fig. 3a, b).

## Discussion

In the present study, we reported that DHRS9 expression was both transcriptionally and translationally downregulated in primary CRC clinical tissues compared with corresponding adjacent normal mucosa tissues. In addition, reduced expression of DHRS9 was significantly correlated with a variety of important clinicopathologic parameters including lymph node metastasis, TNM stage, disease recurrence, and vital status. Finally, survival analysis revealed that lower DHRS9 expression in tumors significantly predicted poorer DFS and DSS and was an independent unfavorable prognostic indicator for CRC. Moreover, combination of DHRS9 with TNM stage had a better survival predictive power than either of the two parameters alone.

Decreased expression of DHRS9 has been documented previously by Jette et al. and Kropotova et al. at the mRNA level [10, 15]. Our real-time qPCR data were also consistent with their findings. Of note, we observed a significant decrease in DHRS9 mRNA level (over twofold) in 65.5 % (38/58) of the cancerous specimens tested, while Jette et al*.* reported that expression of DHRS9 was decreased at least twofold in 90 % (9/10) of carcinoma samples. This discrepancy may be attributed to the different ethnic groups studied, sample size, or endogenous control used for normalization. In addition, our subsequent Western blot assay on the same corresponding samples confirmed that DHRS9 protein was significantly downregulated in 74.1 % (43/58) of the cancerous tissues tested. Furthermore, immunohistochemical analyses of 163 paired CRC samples showed that the immunoreactivity of DHRS9 protein was observed in the cytoplasm, and 91.4 % (149/163) of the normal colorectal mucosa tissues tested were classified as DHRS9-positive, whereas only 57.7 % (94/163) of the cancerous samples were classified as DHRS9-positive. Among them, 46 % (75/163) of the normal mucosa tissues examined were moderate–strong staining of DHRS9 protein, while only 19 % (31/163) of the cancerous tissues showed virtually the same immunoreactivity. Thus, our results definitely confirmed the significant downregulation of DHRS9 expression in CRC at both the mRNA and the protein level. The reported induction of DHRS9 by the tumor suppressor gene APC [15] may, at least partly, explain the reason why DHRS9 expression is reduced in CRC. Nevertheless, the detail mechanism for DHRS9 downregulation in CRC remains to be clarified.

Interestingly, according to our results, decreased expression of DHRS9 protein in CRC was significantly correlated with increased lymph node metastasis, advanced TNM stage, increased disease recurrence, and patient death, indicating that DHRS9 might be negatively involved in the progression of CRC. It is well established that atRA exerts multiple anti-tumor effects by inhibiting proliferation, reducing colony formation, blocking anchorage-independent growth, promoting differentiation, inducing apoptosis, and suppressing invasiveness of cancer cell [12, 22–27], and significantly impaired atRA biosynthesis has been noted in a variety of human malignancies, such as prostate cancer, breast cancer, ovarian cancer, gastric cancer, and CRC [15, 28–31]. Meanwhile, DHRS9 belongs to the atRA-generating enzymes [13, 15, 32], and its reduced mRNA expression has also been observed in CRC tissues and CRC-derived cell lines [10, 15]. Moreover, Jette et al. found that diminished atRA biosynthesis activity was accompanied with decreased DHRS9 expression in a number of CRC-derived cell lines and that reintroduction of the tumor suppressor APC into APC-deficient colon cancer cells not only induced DHRS9 expression but also increased the conversion of retinol to retinoic acid [15]. Therefore, we hypothesized that DHRS9 might, at least partly, influence the progression of CRC through atRA-related pathway. To test this hypothesis, we detected the mRNA expression of DHRS9 and some downstream targets of atRA or CRC cell progression related genes. Wang et al. previously showed that atRA induces the tumor suppressor gene XAF1 expression through an interferon regulatory factor-1 element in colon cancer [33], and Woo et al. recently reported that atRA induces expression of the tumor suppressor gene E-cadherin via inhibition of DNA methylation in HCT116 cells [34]. Interestingly, we found that DHRS9 mRNA expression was positively correlated with XAF1 and E-cadherin mRNA expression in the 58 CRC samples tested (data not shown). These preliminary findings also support a tumor inhibitory role of DHRS9 in human CRC. Nevertheless, it should be noted that whether reduction of DHRS9 indeed contributes to the development and progression of CRC remains to be carefully determined, for the decrease in DHRS9 expression may also be the result of other factors that lead to cancer progression, rather than downregulation of DHRS9 leading to cancer progression. Further in vitro and in vivo studies using gain-of-function as well as loss-of-function strategies are warranted to address this issue.

Another interesting finding of the present study was the prognostic significance of DHRS9 in CRC. Decreased DHRS9 protein expression was significantly associated with shortened DFS and DSS of CRC patients. In univariate analysis, DHRS9 protein emerged as a significant prognostic factor of clinical outcome. Moreover, in multivariate analysis, DHRS9 still emerged as a significant independent predictor of survival in addition to tumor stage. Thus, to our knowledge, the current study is the first to report the prognostic value of DHRS9 in CRC. Notably, our results revealed that TNM stage also is a significant prognostic factor for CRC patients, which is consistent with the well-recognized adverse prognostic effect of tumor stage [35] and confirms that our cohort was representative and that the survival analyses were valid (Table 3 and Fig. 3). More importantly, we demonstrated that combination of DHRS9 protein expression with TNM stage has a more powerful efficiency in prognosis prediction when compared to each of the two parameters alone. Currently, the underlying mechanism for the prognostic importance of DHRS9 expression in CRC is not known and requires further investigation; nevertheless, our data suggest that combination of DHRS9 with TNM stage may have a better prognostic value and could serve as a promising biomarker for classification of CRC patients into distinct risk subgroups and guide individualized therapy choices.

There were several limitations in the present study. Although our results revealed the clinicopathologic correlation and the prognostic value for DHRS9 expression in a cohort of CRC patients, they did not elucidate the role of DHRS9 expression in the development of CRC. Besides, due to the limitation of follow-up period, the median survival time of patients in the high DHRS9 expression group could not be obtained; thus, our current results could not accurately reflect the survival of patients in these subgroups. Additionally, the number of samples in the present study is still relatively small. Therefore, future multi-center, prospective studies using larger cohorts are necessary to verify the robustness of our findings before clinical translation.

In conclusion, we here provided the first evidence that DHRS9 protein expression was frequently downregulated in CRC tissues and that decreased expression of DHRS9 was significantly associated with disease progression and poor outcome of CRC patients. Our data suggest that DHRS9 could be used as a potential prognostic biomarker for CRC and that combination of DHRS9 with TNM stage or other parameters may enhance its performance in prognostic prediction. Apart from its prognostic value, results from the present study encourage further investigation of its potential role in CRC pathobiology.

## Abbreviations

CRC: Colorectal cancer
DHRS9: Dehydrogenase/reductase (SDR family) member 9
DSS: Disease-specific survival
DFS: Disease-free survival
qPCR: Quantitative polymerase chain reaction
IHC: Immunohistochemistry
IRS: Immunoreactive score
RDHL: Retinol dehydrogenase L
APC: Adenomatous polyposis coli
atRA: All-trans-retinoic acid
